# A Snapshot of the Plant Glycated Proteome: STRUCTURAL, FUNCTIONAL, AND MECHANISTIC ASPECTS*

**DOI:** 10.1074/jbc.M115.678581

**Published:** 2016-01-19

**Authors:** Tatiana Bilova, Elena Lukasheva, Dominic Brauch, Uta Greifenhagen, Gagan Paudel, Elena Tarakhovskaya, Nadezhda Frolova, Juliane Mittasch, Gerd Ulrich Balcke, Alain Tissier, Natalia Osmolovskaya, Thomas Vogt, Ludger A. Wessjohann, Claudia Birkemeyer, Carsten Milkowski, Andrej Frolov

**Affiliations:** From the Departments of ‡ Bioorganic Chemistry and; §§ Cell and Metabolic Biology, Leibniz Institute of Plant Biochemistry (IPB), D-06120 Halle (Saale), Germany,; § Faculty of Chemistry and Mineralogy, Universität Leipzig, D-04103 Leipzig, Germany,; Departments of ¶ Biochemistry and; ‖ Plant Physiology and Biochemistry, Faculty of Biology, Saint Petersburg State University, 199034 Saint Petersburg, Russia,; ** Leibniz Institute of Plant Genetics and Crop Plant Research (IPK), D-06466 Stadt Seeland, Germany, and; ‡‡ Interdisciplinary Center for Crop Plant Research (IZN), Martin Luther University Halle-Wittenberg, D-06120 Halle (Saale),Germany

**Keywords:** Arabidopsis thaliana, carbohydrate-binding protein, gas chromatography-mass spectrometry (GC-MS), glycation, mass spectrometry (MS), metabolomics, plant biochemistry, proteomics, Brassica napus, advanced glycation end products (AGEs)

## Abstract

Glycation is the reaction of carbonyl compounds (reducing sugars and α-dicarbonyls) with amino acids, lipids, and proteins, yielding early and advanced glycation end products (AGEs). The AGEs can be formed via degradation of early glycation intermediates (glycoxidation) and by interaction with the products of monosaccharide autoxidation (autoxidative glycosylation). Although formation of these potentially deleterious compounds is well characterized in animal systems and thermally treated foods, only a little information about advanced glycation in plants is available. Thus, the knowledge of the plant AGE patterns and the underlying pathways of their formation are completely missing. To fill this gap, we describe the AGE-modified proteome of *Brassica napus* and characterize individual sites of advanced glycation by the methods of liquid chromatography-based bottom-up proteomics. The modification patterns were complex but reproducible: 789 AGE-modified peptides in 772 proteins were detected in two independent experiments. In contrast, only 168 polypeptides contained early glycated lysines, which did not resemble the sites of advanced glycation. Similar observations were made with *Arabidopsis thaliana*. The absence of the early glycated precursors of the AGE-modified protein residues indicated autoxidative glycosylation, but not glycoxidation, as the major pathway of AGE formation. To prove this assumption and to identify the potential modifying agents, we estimated the reactivity and glycative potential of plant-derived sugars using a model peptide approach and liquid chromatography-mass spectrometry-based techniques. Evaluation of these data sets together with the assessed tissue carbohydrate contents revealed dihydroxyacetone phosphate, glyceraldehyde 3-phosphate, ribulose, erythrose, and sucrose as potential precursors of plant AGEs.

## Introduction

**FIGURE 1.**
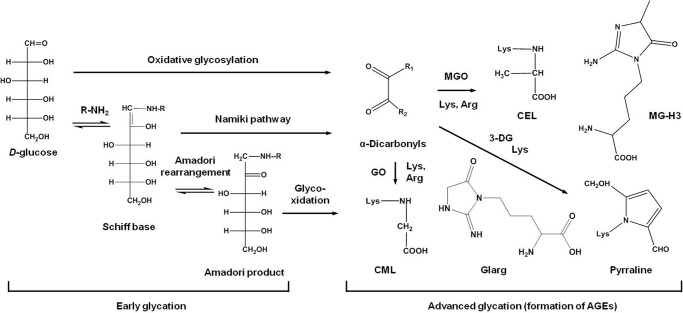
**The principle pathways of early and advanced glycation (monosaccharide autoxidation/oxidative glycosylation, Namiki pathway, and glycoxidation).** *Arg*, protein arginyl residue; *3-DG*, 3-deoxyglycosone; *Lys*, protein lysyl residue; *MG-H3*, 2-amino-5-(2-amino-4-hydro-4-methyl-5-imidazolon-1-yl) pentanoic acid; *R-NH_2_*, side chain or N-terminal group in protein; *R_1_*/*R_2_* is H/H for GO; H/CH_3_ for MGO; and H/C_4_H_9_O_3_ for 3-deoxyglycosone; *Glarg*, glyoxal-derived hydroimidazolone.

Protein glycation is a ubiquitous post-translational modification (1) formed by the reaction of reducing sugars (aldoses and ketoses) with amino groups of lysyl side chains or N termini, yielding imine intermediates further involved in Amadori (2) and Heyns (3) rearrangement, respectively (Fig. 1). Resulting early glycation products (*i.e.* keto- and aldamines, respectively) as well as their Schiff base precursors (Namiki pathway) readily undergo further oxidation (glycoxidation) and cross-linking, yielding a heterogeneous group of advanced glycation end products (AGEs)^3^ (4). Alternatively, free sugars can be involved in metal-catalyzed autoxidation (5), yielding highly reactive α-dicarbonyl compounds glyoxal (GO), methylglyoxal (MGO), and osones (*e.g.* 3-deoxyglucosone) (6). These intermediates can directly react with lysine and arginine side chains of proteins, yielding AGEs (Fig. 1) (7). In mammals, AGEs are markers of aging and atherosclerosis (8) and interact with endothelial or macrophage pattern recognition receptors, activating transcription of proinflammatory molecules via nuclear transcription factor κB (NF-κB)-dependent signal transduction pathways (9).

Recently, protein glycation patterns were comprehensively characterized in human blood by a bottom-up liquid chromatography-tandem mass spectrometry (LC-MS/MS)-based proteomic approach (10, 11). Thereby thousands of proteins were found to be glycated in hyperglycemic (diabetic) and healthy individuals (11–14). These glycation rates can be attributed to high blood glucose concentrations (>4.0 mm) and, therefore, high levels of Amadori product formation and monosaccharide autoxidation, especially in diabetic patients characterized with higher blood sugar concentrations (5). Due to their ionic and/or cross-linking properties, AGEs strongly affect protein structure (15). Thus, glyoxal-derived hydroimidazolone (1-(-4-amino-4-carboxybutyl)-2-imino-5-oxoimidazolidine) and methylglyoxal-derived hydroimidazolone (MG-H) are cationic (16, 17), whereas *N*^ϵ^-carboxymethyl- (CML) and *N*^ϵ^-carboxyethyllysine (CEL) form anions in aqueous solution (18, 19). Moreover, both glyoxal and methylglyoxal form cationic lysine dimers, *i.e.* cross-links (20). As was previously demonstrated in animal models or *in vitro* studies, these changes directly affect specific biological functions: enzyme activities (21), affinity of transport polypeptides to their targets (22), ligand-receptor interaction (23), and mechanical properties of contractile proteins (24).

In contrast to mammals, information on plant glycation is limited, although such systems (rich in highly reactive carbohydrates, *e.g.* fructose, ribose, and arabinose (25)), might have higher glycative potential. Moreover, plant tissues are rich in sugar phosphates, *i.e.* intermediates of glycolysis and the Calvin cycle (26). Also it was demonstrated that triose phosphates (glyceraldehyde 3-phosphate and dihydroxyacetone phosphate) can be non-enzymatically converted to methylglyoxal (27). Obviously, high concentrations of sugars in the presence of transition metal ions ensure high rates of monosaccharide autoxidation, production of reactive oxygen species (ROS), and AGE accumulation (28). The transport of electrons in mitochondria and chloroplasts, photorespiration pathway (especially under conditions of light and drought stress) (28), and ascorbate autoxidation (29) may also impact this process.

Generally, the relevance of glycation in plants is indirectly supported not only by a high activity of ascorbate-glutathione cycle enzymes (30) but also by the existence of multiple antiglycative defense enzymes: glyoxalases I and II (31), ribulosamine/erythrulosamine 3-kinase (26), and acylamino acid-releasing enzyme (32). Recently, in their pioneer work, Bechtold *et al.* (33) detected AGE-modified amino acids in protein hydrolysates obtained from *Arabidopsis thaliana* leaves. However, the plant protein glycation targets and exact modification sites therein are still unknown. Hence, no assumptions concerning physiological effects and mechanisms of plant glycation could be made. Therefore, here we present the first comprehensive in-depth analysis of a glycated proteome from a crop plant species, *Brassica napus*, using *A. thaliana* as a reference organism. Based on the modification patterns and metabolomics data, the pathways of AGE formation and possible glycation agents in plants are proposed. The potential glycation agents of the plant origin were investigated for their relative reactivity *in vitro* and their possible impact on the generation of AGEs *in vivo*.

## Experimental Procedures

### 

#### 

##### Reagents

Unless stated otherwise, materials were obtained from the following manufacturers: Amplite Fluorimetric Glutathione GSH/GSSG Ratio Assay kit, AAT Bioquest, Inc. (Sunnyvale, CA); atmospheric pressure chemical ionization positive calibration solution for the AB Sciex Triple TOF^TM^ Systems and polypropylene glycol solutions, AB Sciex (Darmstadt, Germany); Tris (ultrapure), AppliChem GmbH (Darmstadt, Germany); acetonitrile (MS grade), *N*,*N*′-dimethylformamide (≥99.8%), piperidine (≥99.5%), and dichloromethane (≥99.9%), Biosolve (Valkenswaard, Netherlands); dithiothreitol (DTT; ≥99.9%), trifluoroacetic acid (TFA; ≥99.9%), glacial acetic acid, methanol (≥99.9%), d-fructose (≥99.5%), dimethyl sulfide (pro analysis), α-d-glucose monohydrate (≥99.5%), sodium dodecylsulfate (≥99.5%), d-saccharose (≥99.5%), tris (2-carboxyethyl)phosphine hydrochloride (≥98%), glycerin (≥99.5%), and silica gel 60, Carl Roth GmbH and Co. KG (Karlsruhe, Germany); hexane (puriss), Conlac GmbH (Leipzig, Germany); 9-fluorenylmethoxycarbonyl (Fmoc) Rink Amide AM resin (peptide synthesis grade), Iris Biotech GmbH (Marktredwitz, Germany); Fmoc-protected l-amino acids (peptide synthesis grade), ORPEGEN Pharma (Heidelberg, Germany); porcine trypsin (premium grade), Promega GmbH (Mannheim, Germany); d-ribulose, d-erythrose, and d-3-phosphoglyceric acid disodium salt, Santa Cruz Biotechnology, Inc. (Heidelberg, Germany); acrylamide/bisacrylamide solution (37.5:1, 30% (w/v), 2.6% C), ammonium persulfate (pro analysis), *N*,*N*,*N*′,*N*′-tetramethylethane-1,2-diamine (research grade), Coomassie Brilliant Blue G-250 (pure), glycine (pro analysis), and sucrose (>98%), SERVA Electrophoresis GmbH (Heidelberg, Germany); ethyl acetate (puriss), Solvadis GmbH (Frankfurt am Main, Germany); diethyl ether (100%) and acetonitrile (≥99.9%), VWR International GmbH (Dresden, Germany). All other chemicals were purchased from Sigma-Aldrich Chemie GmbH. Water was purified in house (resistance >18 megaohms/cm; total organic content <1 ppb) on a PureLab Ultra Analytic System (ELGA Lab Water, Celle, Germany).

##### Peptide Synthesis

The sequences of the model peptides were selected with respect to considerations summarized elsewhere (7, 34). Peptides Ac-AFGSAKASGA-NH_2_ (**1**) and Ac-AFGSARASGA-NH_2_ (**2**) as well as other sequences of variable length and amino acid composition (H-AKASASFL-NH_2_, H-AKASAHFL-NH_2_, H-AKASAEFL-NH_2_, Ac-ASSGAGSSARASSGAASSA-NH_2_, Ac-ASSGAGSSARASSGHASSA-NH_2_, and Ac-ASSGAGSSARASSGEASSA-NH_2_ (**3**-**8**, respectively)), were synthesized on a Syro2000 multiple peptide synthesizer (MultiSynTech GmbH, Witten, Germany) by Fmoc/*tert*-butyl chemistry using 8 eq of Fmoc-amino acid derivatives activated with *N*,*N*′-diisopropylcarbodiimide/1-hydroxybenzotriazole in *N*,*N*′-dimethylformamide (35). The peptidyl resins were washed with dichloromethane, dried, and cleaved with TFA containing 12.5% (v/v) scavenger mixture (ethane dithiol, *m*-cresol, thioanisole, and water, 1:2:2:2 by volume). After 2 h the peptides were precipitated and washed twice with cold diethyl ether, dried, and purified on a C_18_ column using a linear aqueous acetonitrile gradient in the presence of 0.1% TFA (v/v) as an ion-pairing reagent.

##### In Vitro Glycation

Synthetic peptides (25 nmol) were dissolved in 0.1 m sodium phosphate buffer, pH 7.4 (50 μl) containing 18 μm FeSO_4_ and one of the following carbohydrates (2.5, 25, and 250 nmol): d-glucose (Glc), d-glucose 6-phosphate (Glc-P), d-fructose (Fru), d-fructose 6-phosphate (Fru-P), d-fructose 1,6-bisphosphate, d-maltose (Mal), d-sucrose (Suc), galactinol dehydrate (Gal-ol), d-raffinose, l-ascorbate (Asc), d-ribose (Rib), d-ribose 5-phosphate, d-ribulose (Rul), d-ribulose-5-phosphate, d-ribulose 1,5-bisphosphate, d-erythrose (Ery), d-erythrose 4-phosphate, d-3-phosphoglyceric acid, d-glyceraldehyde 3-phosphate (Glal-P), and dihydroxyacetone phosphate (Dap). The mixtures were incubated at 22 °C with continuous shaking (60 rpm) in an incubator (3032, GFL GmbH, Burgwedel, Germany). The effects of neighboring residues on glycation reaction were addressed under the same conditions using well established model peptides **3**-**8**. The incubations with highly abundant plant sugars were performed with 10 nmol of individual peptides **1** and **2** in 100 ml of phosphate buffer using sugar/peptide ratios of 792:1 (Glc), 692:1 (sorbose), 591:1 (Suc), 580:1 (Fru), 78:1 (inositol), 3:1 (d-mannose, Man), 2:1 (Rib), 1.7:1 (d-galactose, Gal), 1.1:1 (Mal), and 1:1 (d-xylose). Reference incubations were performed under the same conditions using a 1:1 sugar/peptide ratio. All glycation reactions were stopped immediately after mixing of components (time point 0) or 42 days later by addition of aqueous diethylene triamine pentaacetic acid (6 mm; 10 μl) on ice and stored at −80 °C until further analysis.

##### Plant Material and Cultivation

Spring oilseed rape (*B. napus*, cultivar “Lisora”) was obtained from Norddeutsche Pflanzenzucht (Holtsee, Germany). Seeds were sown onto wet filter paper and germinated for 2 days in the dark at 19 °C in covered growth chambers providing saturating humidity. The seedlings were kept for 5 days in the growth chambers under greenhouse conditions characterized by a 16-h light/8-h dark cycle at 22 °C day and 18 °C night temperatures. Seedlings were then transferred to 2.5-liter polyethylene pots (Auer GmbH, Amerang, Germany) covered with aluminum foil and filled with 1:10 diluted (minimal) culture medium. Thereby five seedlings were inserted in truncated (5–7-mm) 1-ml polypropylene pipette tips and fixed in each pot cover. The pots were continuously supplied with air by means of an air pump (300 liters/h, 4.5 watts; Tetratec 300, Zooplus AG, München, Germany) connected to pots by silicon tubing (5-mm inner diameter/8-mm outer diameter; Labmarket GmbH, Mannheim, Germany) and 5-cm needles (B. Braun Melsungen AG, Melsungen, Germany). After growing for 3 days in the greenhouse under the same light/temperature regime and about 60% humidity, only one of the five plantlets was left in each pot, and the full culture medium (0.90 g/liter Ca(NO_3_)_2_ × 4H_2_O, 0.65 g/liter KNO_3_, 0.07 g/liter NH_4_NO_3_, 0.20 g/liter MgSO_4_ × 7H_2_O, 0.25 g/liter KH_2_PO_4_, 0.0029 g/liter H_3_BO_3_, 0.0019 g/liter MnSO_4_ × 5H_2_O, 0.0002 g/liter ZnSO_4_ × 7H_2_O, 0.0002 g/liter CuSO_4_ x 5H_2_O, 0.0001 g/liter (NH_4_)Mo_7_O_24_ × 4H_2_O, 0.025 g/liter FeSO_4_ × 7H_2_O, 0.032 g/liter Na_2_EDTA × 2H_2_O) was provided 4 days later. The plants were grown for a further 28 days with one change of medium after 14 days. *A. thaliana* (Columbia 1092) seeds were planted in wet soil-sand mixture, and the plants were grown in a phytotron MLR-351H (Sanyo Electric Co., Ltd., Moriguchi, Japan) under a short day with a 8-h light (150 ± 2.5 m photons m^−2^ s^−1^)/16-h dark cycle at 23/18 °C, respectively, and 60% humidity. The plants were harvested, and the leaves were ground in liquid nitrogen using a Mixer Mill MM 400 ball mill with 3-mm-diameter stainless steel balls (Retsch, Haan, Germany) at a vibration frequency of 30 Hz for 2 min. Plant material was obtained from two independent cultivations(*n* = 2).

##### Biochemical Analyses

Lipid hydroperoxides were quantified in leaf tissue by oxidation of a Fe(II)-xylenol orange complex as described by Griffiths *et al.* (36), whereas Asc and dehydroascorbic acid (DHA) were determined according to Huang *et al.* (37). To determine the contents of reduced and oxidized glutathione (GSH and GSSG, respectively), ∼10 mg of frozen plant material was left for 3 min on ice before extraction with 250 μl of ice-cold 7% (w/v) 5-sulfosalicylic acid. The samples were vortexed for 30 s and centrifuged at 20,000 × *g* for 10 min at 4 °C. The supernatants were neutralized with saturated aq. Na_2_CO_3_ and diluted 3-fold with the “sample buffer” provided in the Amplite Fluorimetric Glutathione GSH/GSSG Ratio Assay kit. GSH and GSSG were quantified according to the manufacturer's instructions.

##### Protein Isolation, Determination of Glyoxalase II Activity, and Tryptic Digestion

Approximately 200 mg of frozen plant material was combined with 1.5 ml of ice-cold isolation buffer (1 mm MgCl_2_, 150 mm NaCl, 0.5 mol/liter EDTA, 5 mm DTT in 20 mm potassium phosphate buffer, pH 8.0). The suspension was vortexed for 30 s and centrifuged for 10 min at 21,000 × *g*. The supernatant (1.25 ml) was collected; the pellet was resuspended in 1.5 ml of the fresh ice-cold isolation buffer, vortexed for 30 s, and centrifuged for 10 min at 21,000 × *g*; and the supernatant (1.25 ml) was combined with the first portion. The extracts were purified by gel filtration chromatography using protein desalting PD-10 columns (GE Healthcare Europe GmbH) equilibrated with 25 mm aq. NH_4_HCO_3_. The eluate (3.5 ml) was ultrafiltrated against the same buffer (3 × 2 ml) and concentrated by a Vivaspin sample concentrator (molecular weight cutoff, 10,000; Sartorius Mechatronics T&H GmbH, Hamburg, Germany) to a volume of 200 μl. The protein concentrations were determined by the Bradford assay with BSA as the standard as described by Frolov *et al.* (38). For determination of the glyoxalase II activity, 50 μl of protein extracts was added to a mixture of 0.2 mm 5,5′-dithiobis(2-nitrobenzoic acid) and 1 mm *S*-d-lactoylglutathione (450 μl), and absorption (412 nm) was determined spectrophotometrically for 3 min with 30-s intervals. Alternatively, the protein aliquots (200 μg) were diluted to 207.4 μl with 25 mm NH_4_HCO_3_ and digested with trypsin as described by Bollineni *et al.* (39). The efficiency of digestion was verified by SDS-PAGE as described elsewhere (12).

##### Boronic Acid Affinity Chromatography (BAC)

BAC was performed as described by Frolov and Hoffmann (40) with changes. *m*-Aminophenylboronic acid-agarose (1 ml) was resuspended in aq. 250 mm NH_4_CH_3_COO, 50 mm Mg(CH_3_COO)_2_ × 6 H_2_O, pH 8.1, and packed in a 1-ml polypropylene column (85 × 7.5 mm; Qiagen GmbH, Hilden, Germany). 200 μg of the lyophilized tryptic digest was reconstituted in 20% (v/v) aq. CH_3_CN and diluted with ice-cold washing buffer to a final volume of 500 μl. After washing out the unbound fraction with 13.5 ml of loading buffer, glycated peptides were eluted in two steps with aq. CH_3_COOH (0.1 m, 8 ml; then 0.2 m, 2 ml) at 37 °C. Eluates were lyophilized and stored at −20 °C until further analysis.

##### Solid Phase Extraction

The digests or BAC eluates (reconstituted in 300 μl of 3% (v/v) CH_3_CN in 0.1% (v/v) HCOOH) were loaded on Oasis HLB cartridges (10 mg, 30 μm; Waters GmbH, Eschborn, Germany) using a Chromabond vacuum manifold (Machery Nagel, Düren, Germany). After washing with 1 ml 0.1% (v/v) aq. formic acid, the elution from the cartridges was performed with 333 μl of 40, 60, and 80% (v/v) aq. CH_3_CN in 0.1% (v/v) HCOOH. The combined eluates were concentrated under vacuum for 30 min, freeze-dried, and stored at −20 °C.

##### Gas Chromatography-Mass Spectrometry (GC-MS)

GC-MSanalyses were performed on an Agilent 6890 gas chromatograph (1 ml/min helium flow; splitless mode; injector temperature, 250 °C) coupled to an Agilent 5973 quadrupole mass-selective detector (Agilent Technologies, Böblingen, Germany).The electron ionization spectra were acquired at 70 eV.

For analysis of GO and MGO, 700 μl of water was added to 100 mg of frozen plant material in a 2-ml polypropylene tube. The suspension was vortexed for 30 s, and 5 μl of 100 mm *O*-(2,3,4,5,6-pentafluorobenzyl) hydroxylamine were added. After sonication for 5 min, perchloric acid (2 m; 175 μl) was added, and the samples were incubated for 60 min at 40 °C while continuously shaking (1000 rpm). After acidification with sulfuric acid (7 m; 10 μl), the *O*-(2,3,4,5,6-pentafluorobenzyl) hydroxylamine derivatives were extracted three times with 200 μl of cyclohexane. The combined extracts were dried under reduced pressure and reconstituted in 60 μl of cyclohexane containing 0.01% suberic acid dimethyl ester (internal standard), and 1 μl of each sample was injected (2-min splitless time) onto a (35% phenyl)-methylpolysiloxane mid-polarity DB-35 MS column (30 m × 0.25-mm inner diameter, 0.25-μm film thickness; Agilent J&W, Waldbronn, Germany) by a 10 °C/min ramp from 50 to 325 °C followed by 15 min at 325 °C. The spectra were acquired in the *m*/*z* range of 50–800 with a scan rate of 0.5 s/scan. Quantitation relied on the specific signals of the bisglyoxal and bismethylglyoxal *O*-(2,3,4,5,6-pentafluorobenzyl) hydroxylamine derivatives (*m*/*z* 448 and 462, respectively) and standard addition with five concentration levels.

For the analysis of carbohydrates, 20 mg of frozen plant material was placed in a 2-ml polypropylene tube and supplemented with 800 μl of methanol. After vortexing (30 s) and centrifugation (15,000 × *g*, 1 min, 4 °C), 500 μl of supernatant was transferred to a new tube before 400 μl of deionized water was added to the residues, and extraction was repeated. After centrifugation (15,000 × *g*, 1 min, 4 °C), 600 μl of the supernatant was removed and combined with the first portion. After lipid extraction (0.5 ml of *n*-hexane), 110 μl of the polar extract was dried under reduced pressure and derivatized as described by Milkovska-Stamenova *et al.* (41), and 1 μl of each sample was separated on an HP-5 Trx-5 MS capillary column (30 m × 0.25-mm inner diameter, 0.25-μm film thickness; Thermo Fisher Scientific, Bremen, Germany) by sequential 15 and 6 °C/min ramps from 40 to 70 °C and from 70 to 320 °C, respectively, followed by 10 min at 320 °C (1.5-min splitless time). The spectra were acquired in the *m*/*z* range of 50–550 with a scan rate of 0.34 s/scan. Identification relied on retention time indices and a spectral similarity search (NIST08 and in-house libraries), whereas quantitation was based on integration of the corresponding extracted ion chromatograms (XICs; ±0.5 Da) at specific retention times (*t_R_*). Calibration was performed by the standard addition method using five calibration levels.

##### Electrospray Ionization (ESI)-Orbitrap-Linear Ion Trap (LIT)-MS and MS/MS

Tryptic digests (70 ng) were loaded on a nanoAcquity UPLC® Symmetry trap column (C_18_ phase; 0.18 × 20 mm; particle size, 5 μm; Waters GmbH) and separated on a nanoAcquity UPLC bridged ethylene hybrid BEH130 column (C_18_ phase; 0.1 × 100 mm; particle size, 1.7 μm) using a nanoAcquity UPLC System controlled by MassLynx X.4.1 software (Waters GmbH). Eluents A and B were water and acetonitrile both containing 0.1% (v/v) formic acid. After injection (10 μl; full loop mode), the peptides were trapped for 5 min at 5 μl/min and, after a 5 min isocratic step at 3% eluent B, separated in sequential linear gradients to 40 and 85% eluent B in 45 and 2 min, respectively. After a 5-min wash at 85% eluent B, the column was re-equilibrated for 10 min with 3% eluent B. The separations were performed at 30 °C and a flow rate of 0.4 μl/min. The column effluents were transferred via a PicoTip on-line nano-ESI emitter to an LTQ Orbitrap XL electron transfer dissociation mass spectrometer operating in positive ion mode equipped with a nano-ESI source (ion spray voltage, 1.5 kV; capillary temperature, 200 °C) and controlled by Xcalibur 2.0.7 software (Thermo Fisher Scientific). Analyses relied on a survey Orbitrap scan followed by collision-induced dissociation fragmentation in the LIT in data-dependent acquisition (DDA) mode for the six most intense signals with charge states from 2 to 5. The Orbitrap scans were acquired at *m*/*z* ranges of 400–600, 600–800, and 800–1500 at a resolution of 30,000. The LIT scans were acquired with 30% normalized collision energy. Identification of peptide sequences and protein annotation relied on the Sequest search against an *A. thaliana* protein database (*A. thaliana* proteome reviewed, UniProt, October 14, 2013) using modification-specific mass increments and result filtering as described elsewhere (12). Annotation of protein functions was performed with the MapMan software (Max Planck Institute of Molecular Plant Physiology, Potsdam-Golm, Germany; June 9, 2014).

##### ESI-Quadrupole-Time of Flight (QqTOF)-MS and MS/MS

The incubation mixtures (50 pmol of the peptide equivalent) were loaded on an Acquity UPLC reversed phase charged surface hybrid CSH^TM^ column (C_18_ phase; inner diameter, 0.1 mm; length, 50 mm; particle size, 1.7 μm) and separated in sequential linear gradients from 3 to 7, 16, and 95% eluent B in 0.5, 4.0, and 0.5 min, respectively. Separation was performed on an Acquity UPLC System with a flow rate of 0.3 ml/min and 45 °C column temperature using eluents A and B identical to those in the previous section. The column effluent was introduced on line into a TripleTOF 5600-1 QqTOF mass spectrometer equipped with a DuoSpray ESI/atmospheric pressure chemical ionization ion source operating in positive ion mode and controlled by Analyst TF 1.6 software (AB Sciex). The spectra were acquired in the *m*/*z* range of 350–1300 (pulser frequency, 16.38 Hz) with an ion spray voltage of 5.5 kV and 450 °C source temperature. Nebulizer and drying gases were set to 60 and 70 p.s.i., respectively. MS/MS spectra were acquired at the *m*/*z* range of 50–1300 using collision potentials of 30–110 V and 20-V collision energy spread. Peak alignment and preliminary statistical evaluation were performed by MarkerView 1.2.4 software, whereas MultiQuant 2.02 software (AB Sciex) was applied for peak integration.

## Results

### 

#### 

##### Plant Growth and Characterization

**FIGURE 2.**
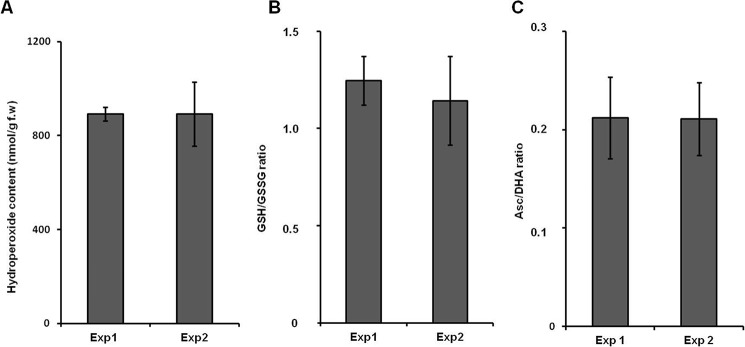
**Characterization of plant redox status in two independent experiments (*Exp1* and *Exp2*) by determination of lipid hydroperoxide contents (*A*), GSH/GSSG ratio (*B*), and Asc/DHA ratio (*C*).** The analyses relied on the ferrous oxidation-xylenol orange assay with absorption measurement at 650 nm (*A*), fluorescence measurement at an excitation/emission wavelength of 490/520 nm after specific derivatization using the Amplite Fluorimetric Glutathione GSH/GSSG Ratio Assay kit (*B*), and the ascorbate oxidase method using detection of DHA absorption at 265 nm (*C*). Data are means with error bars representing S.D.

After 1 week of growth on the wet filter paper, most of the *B. napus* seedlings developed cotyledons and main roots of at least 3 cm in length. Thus, the seedlings could be easily transferred to the pots, and only minimal plant losses were observed during this step (≤5%). After transfer to the pots and removal of the four neighboring seedlings 3 days later, all plants developed shoots of comparable size during the following 3 weeks of growth. After the 6th week, the plants reached the size of at least 40 cm with shoot and root fresh weights of 118 ± 20 and 43 ± 9 g, respectively. No visible morphological differences between the two cultivation experiments were observed. The stress status of the plants was also comparable as no differences in the contents of lipid hydroperoxides, as well as Asc/DHA and GSH/GSSG ratios between two experiment replicates, were observed (Fig. 2). The reference plants (*A. thaliana*) had developed rosettes of 6–8-cm diameter and 0.5–1-g weight. In comparison with *B. napus*, the plants showed essential differences in the parameters of oxidative stress. Thus, although the GSH/GSSG ratios were comparable in both plants (1.41 ± 0.18), the Asc/DHA ratios and lipid hydroperoxide contents were higher in *A. thaliana* (1.34 ± 0.09 and 1.58 ± 0.08 μmol/g fresh weight (f.w.), respectively).

##### Protein Isolation and Tryptic Digestion

As the protein isolation workflow comprised gel filtration chromatography and ultrafiltration, we estimated the protein losses in both purification steps. Therefore, the protein contents determined directly after the extraction were defined as 100%. The results indicated ∼10 and 35% protein losses in gel filtration chromatography and ultrafiltration, respectively, resulting in an overall recovery of 58 ± 7%. The overall protein yields were in the range of 1.43–2.01 mg/g f.w. The tryptic digestion was complete as the ribulose bisphosphate carboxylase/oxidase (Rubisco) large subunit band was not detectable (that was the case with undigested protein extracts), indicating a digest efficiency >95% and a method sensitivity better than 80 ng/band (38) with Rubisco content representing at least 20% of the total leaf protein weight (42).

##### Characterization of the AGE-modified Proteome

In *B. napus*,the DDA experiments complemented with a gas phase fractionation approach and *t_R_*-based exclusion of non-modified peptides (*i.e.* previously identified peptides are excluded from fragmentation in a second analysis by implementing their *m*/*z* and a corresponding retention time window in an exclusion list into the instrument methods) revealed 900 AGE-containing peptides representing 1264 glycation sites in 883 proteins detected overall in two experimental replicates. The most abundant of them was the large subunit of Rubisco, whereas the 187-kDa microtubule-associated protein AIR9, mediator of RNA polymerase II transcription subunit 12, phosphatidylinositol *N*-acetylglucosaminyltransferase subunit P-like protein, chloroplastic ATP synthase gγ chain 2, fatty acyl-CoA reductase 8, chloroplastic peptidyl-prolyl *cis-trans* isomerase CYP20-3, and proteasome subunit α type 5-A were of the lowest quantities. Comparison of the database searches performed for each replicate separately revealed 789 peptides (containing 1082 AGE modification sites in 772 proteins) in common for both data sets. Thus, 85 and 87% of the AGE-modified proteome characterized in experiments 1 and 2, respectively, were reproducibly detected. However, 64 and 47 proteins detected in experiments 1 and 2, respectively, were experiment-specific. The peptide signals representing these molecules had intensities close to a signal/noise ratio of 3, which could be the reason of their absence in the other replicate.

**FIGURE 3.**
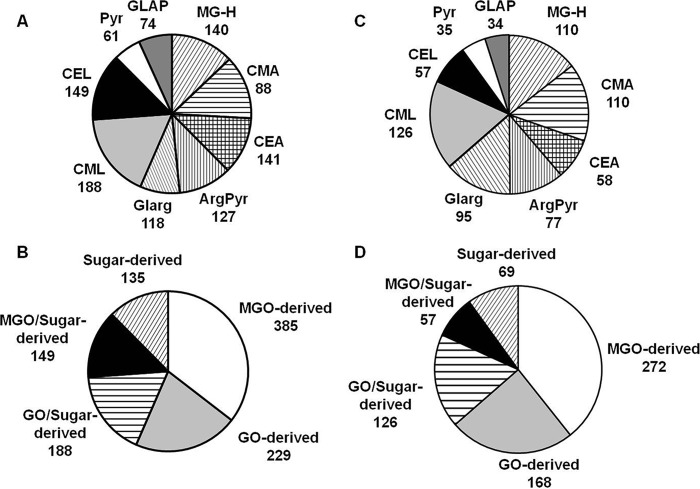
**AGE-modified tryptic peptides obtained from *B. napus* (*A* and *B*) and *A. thaliana* (*C* and *D*) soluble proteins distributed by classes of AGE modifications (*A* and *C*) and metabolic origin (*B* and *D*).** The AGE patterns were identified by nanoscale UPLC-ESI-Orbitrap-LIT-MS operated in positive DDA mode. Glycation sites were identified by database search using the Sequest engine. *ArgPyr*, argpyrimidine; *CMA*, *N*^ϵ^-carboxymethylarginine; *GLAP*, glyceraldehyde-derived pyridinium compound; *Glarg*, glyoxal-derived hydroimidazolone; *Pyr*, pyrraline.

**FIGURE 4.**
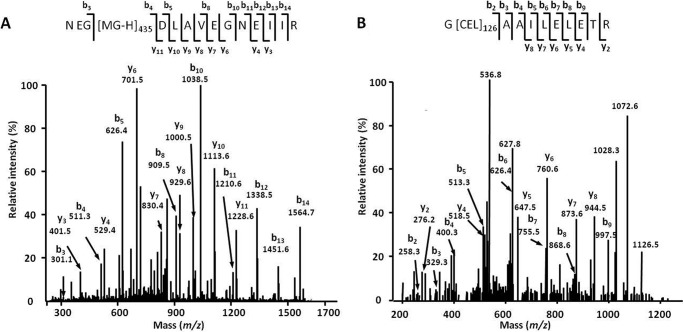
**Tandem mass spectra obtained for *m*/*z* 869. 94 (peptide NEG[MG-H]_435_DLAVEGNEIIR, large chain of Rubisco, EC 4.1.1.39; *A*) and *m*/*z* 636.86 (peptide G[CEL]_126_AAILELETR, enoyl-CoA hydratase 2, EC 4.2.1.119; *B*).** The spectra were acquired by nanoscale UPLC-ESI-Orbitrap-LIT-MS operated in positive DDA mode. The peptide sequences were derived by database search and confirmed by manual interpretation. *b_n_*, N-terminal fragment ion series; *y_n_*, C-terminal fragment ion series; *subscripts 435* and *126* represent the positions of MG-H and CEL in the corresponding polypeptide backbones.

Peptide sequence assessment and post-translational modification localization relied on b- and y-fragment ion series, which are typically abundant for confidently identified species (Fig. 3). The modification sites were mostly represented by arginines (614 sites; *i.e.* 57% of identified AGE residues; Fig. 4*A*) and dominated by MG-H and *N*^δ^-(carboxyethyl)arginine (CEA). The distribution of the individual AGEs favored the MGO-derived residues (385 residues, or 49%) followed by 229 (29%) GO-derived residues, whereas only a minor portion, represented by pyrraline and glyceraldehyde-derived pyridinium compound (135 residues, or 17%) were the products of interaction with free carbohydrates as well as degradation of corresponding Amadori or Heyns compounds (Fig. 4*B*). Forty-two percent of the glycation sites could have both α-dicarbonyls and early glycated products as precursors (CML and CEL).

To address the intraspecies differences in glycation patterns, the acquired data were compared with those obtained with *A. thaliana*, which was taken as a reference plant. Thus, 520 AGE-modified peptides representing 692 glycation sites in 502 proteins were identified in the *A. thaliana* proteome. Among these sites, 440 (64%) and 252 (36%) were represented by arginyl and lysyl residues, respectively. Thereby CML, MG-H, and CEA dominated in the AGE patterns (Fig. 4*C*). Most of the glycated residues originated from MGO (272; 39%) followed by GO- (168; 24%) and sugar/early glycation product-derived residues (69; 10%), whereas 183 residues (26%) could originate from several precursors (Fig. 4*D*).

Functional annotation of the AGE-modified polypeptides clearly indicated those related to protein and RNA metabolism (116 and 78 entries, respectively) as the most frequently affected groups (Table 1). The proteins related to transport, cell regulation, DNA synthesis, and enzymatic catalysis were also AGE-modified (25–36 entries for each group). No less important was the observed interference of advanced glycation with the pathways of antioxidative and antiglycative defense. For instance, such important mediators of ROS homeostasis as peroxidases 4 and 64, ferric reduction oxidase, glutathione *S*-transferase, and glyoxal oxidase were found to be AGE-modified. Moreover, the proteins involved in hormonal response (*e.g.* ethylene-responsive transcription factors GRF5 and ERF084, abscisic-aldehyde oxidase, and auxin-responsive family protein), probably impacting adaptation to environmental stress, were modified at arginyl residues.

**TABLE 1:** **Functional annotation of *B. napus* and *A. thaliana* proteins based on the TAIR9 database** To assign the functions, the protein accession numbers annotated in the proteomic experiments were processed by MapMan software (Max Planck Institute of Molecular Plant Physiology; June 9, 2014). AA, amino acid.

Protein functional groups	Percentage of the database*^a^*	Percentage of AGE-modified		Percentage of Amadori/Heyns-modified	
		in *B. napus**^b^*	in *A. thaliana**^c^*	in *B. napus**^d^*	in *A. thaliana**^e^*
Photosynthesis	0.6	0.9	4.8	Not found	1.8
Sugar metabolism	0.4	1.6	2.8	Not found	3.7
Glycolysis/glyoxylate	0.2	0.4	0.0	Not found	1.8
Electron transport/redox	0.6	0.8	0.6	Not found	Not found
Lipid metabolism	1.2	2.1	1.4	0.6	4.3
Nitrogen/sulfur metabolism	0.1	0.3	Not found	Not found	Not found
AA metabolism	1.0	0.9	3.6	Not found	Not found
Protein metabolism	13.6	15.4	49.2	5.4	47.6
Secondary metabolism	1.4	1.5	2.8	Not found	1.2
Hormone metabolism	1.6	2.0	3.2	0.6	3.0
Miscellaneous	4.7	4.0	9.0	1.2	Not found
RNA processing/regulation of transcription	9.0	10.4	29.1	5.4	22.6
DNA synthesis/repair	8.5	4.8	9.6	1.2	14.6
Signaling	4.0	4.6	13.7	1.8	14.6
Cell	4.0	4.2	9.2	0.6	7.9
Development	3.9	1.7	5.2	Not found	2.4
Transport	3.9	3.3	2.8	1.8	4.9
Organ transformation	0.2	0.5	0.4	1.2	Not found
Cell wall	3.9	0.8	5.0	Not found	1.8
Stress	3.6	3.7	8.6	0.6	17.1
Metal handling	1.1	0.5	Not found	Not found	Not found
Not assigned	34.6	34.5	60.6	12.5	31.1

*^a^* Percentage of all TAIR9 database entries (34,300 entries) represented by individual protein functional groups.

*^b^* Percentage of all proteins identified as AGE-modified in both experiments with *B. napus* (753 entries).

*^c^* Percentage of all proteins identified as AGE-modified in both experiments with *A. thaliana* (502 entries).

*^d^* Percentage of all proteins identified as early glycated in *B. napus* (753 entries).

*^e^* Percentage of all proteins identified as early glycated in *A. thaliana* (502 entries).

##### Potential Precursors of AGE Formation in Plants

**FIGURE 5.**
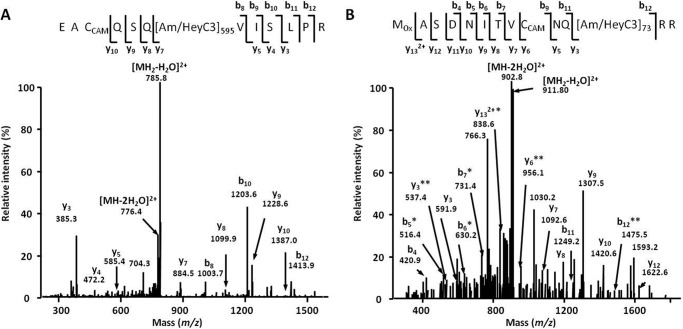
**Tandem mass spectra obtained for *m*/*z* 794. 41 (triose-modified peptide EAC_CAM_QSQ[Am/HeyC3]_595_VISLPR, chloride channel protein CLC-d; *A*) and *m*/*z* 920.94 (pentose-modified peptide M_Ox_ASDNITVC_CAM_NQ[Am/HeyC3]_73_RR, cyclin-A2-1; *B*).** The spectra were acquired by nanoscale UPLC-ESI-Orbitrap-LIT-MS operated in positive DDA mode. The peptide sequences were derived by database search and confirmed by manual interpretation. *Am/HeyC3*, triose-derived Amadori or Heyns product on a lysyl residue; *b_n_*, N-terminal fragment ion series; *C_CAM_*, carbamidomethylated cysteine; *M_Ox_*, oxidized methionine; *y_n_*, C-terminal fragment ion series. * (−H_2_O) and ** (−2 H_2_O) indicate water losses. Water losses (one or two molecules) from the quasimolecular ion are indicated as *[MH_2_ − H_2_O]*^*2*+^ and *[MH_2_ − H_2_O]*^*2*+^, respectively. *Subscripts 595* and *73* represent position of Am/HeyC3 in corresponding polypeptide backbones.

**FIGURE 6.**
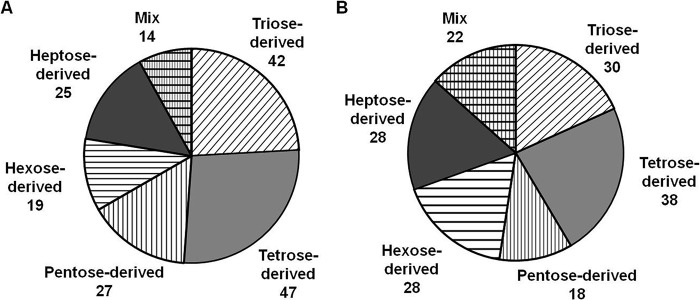
**Amadori/Heyns peptides from *B. napus* (*A*) and *A. thaliana* (*B*) identified by characteristic MS/MS fragmentation patterns and *m*/*z* increments of precursor and fragment signals (Δ*m*) of 72 mass units for triose-derived, 102 mass units for tetrose-derived, 132 mass units for pentose-derived, 162 mass units for hexose-derived, and 192 mass units for heptose-derived products as well as their combination (*Mix*).** The glycation patterns were identified in BAC eluates by nanoscale UPLC-ESI-Orbitrap-LIT-MS operated in positive DDA mode. Glycation sites were identified by database search using the Sequest engine.

To address the possible origin of the detected AGEs, we assessed the levels of early protein glycation (*i.e.* the number and distribution of keto- and aldamine modifications) as well as the tissue contents of GO, MGO, and carbohydrates. The analysis of BAC eluates obtained from pooled *B. napus* digests revealed 176 glycated peptides representing 188 modification sites in 168 proteins. None of these sites was found to be AGE-modified. The peptide sequences, exact positions of the Amadori/Heyns moieties, and their elemental composition could be unambiguously assigned by characteristic MS/MS fragmentation patterns (Fig. 5), although the exact structural assignment of the sugar moiety (Amadori or Heyns product) was not possible. The early glycation patterns were dominated by triose- and tetrose-derived modifications. Remarkably, the hexose-derived modifications (highly abundant in mammals (11)) were much less abundant (Fig. 6*A*). These data were very similar to those obtained with the reference plant (*A. thaliana*) where 162 proteins represented by 164 peptides containing an overall 198 sites could be identified as early glycated with ∼50% sites being triose-and tetrose-modified (Fig. 6*B*). Functional annotation of the early glycated *B. napus* proteome revealed 15 protein groups dominated by those responsible for the regulation of RNA synthesis and transcription (5.4% from the total number of modification sites; Table 1). The other physiological functions were much less affected: maximally three modification sites could be assigned to each additional group. This was in agreement with the *Arabidopsis* data, indicating the polypeptides responsible for protein synthesis and RNA metabolism as the most glycated (47.6 and 22.6% of total identified early glycation sites, respectively; Table 1).

The tissue contents of GO and MGO were comparable for the first and second experimental replicates. The contents of GO in *B. napus* tissues (4.49 ± 1.055 and 3.07 ± 0.736 nmol/g f.w., respectively) were 3-fold higher in comparison with MGO (1.51 ± 0.18 and 0.88 ± 0.13 nmol/g f.w., respectively). These differences were statistically significant (*t* test; *p* = 0.020 and 0.019 for the first and second experiments, respectively). The GO and MGO contents in *A. thaliana* tissues were 2.70 ± 0.68 and 0.85 ± 0.14 nmol/g f.w., respectively. At the same time, the activity of glyoxalase II was ∼2.5 times higher in *A. thaliana* leaf tissue in comparison with *B. napus* (0.42 ± 0.05 and 0.17 ± 0.004 μmol min^−1^ g^−1^ f.w., respectively).

**FIGURE 7.**
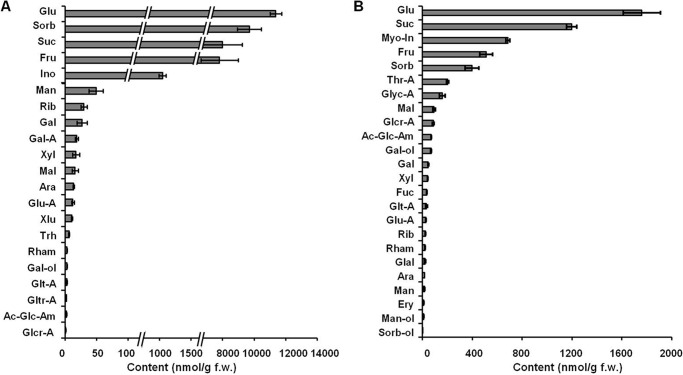
**The contents of individual carbohydrates in *B. napus* (*A*) and *A. thaliana* (*B*) leaf methanolic extracts determined by GC-electron ionization-quadrupole-MS after sequential derivatization with methoxyamine hydrochloride in pyridine and *N*-methyl-*N*-(trimethylsilyl) trifluoroacetamide.** Quantification relied on the standard addition technique (*B. napus*) or external standardization (*A. thaliana*). *Ac-Glc-Am*, *N-*acetyl-d-glucosamine; *Ara*, arabinose; *Fuc*, fucose; *Gal-A*, galactonic acid; *Glal*, d-glyceraldehyde; *Glcr-A*, glucaric acid; *Glt-A*, galactaric acid; *Gltr-A*, galacturonic acid; *Glyc-A*, glyceric acid; *Glu-A*, gluconic acid; *Ino*, inositol; *Man-ol*, mannitol; *Myo-in*, myoinositol; *Rham*, rhamnose; *Sorb*, sorbose; *Sorb-ol*, sorbitol; *Thr-A*, threonic acid; *Trh*, trehalose; *Xlu*, xylulose; *Xyl*, d-xylose. Data are means with error bars representing S.D.

The GC-electron ionization-quadrupole-MS analysis revealed the presence of 21 carbohydrates in plant tissues (Fig. 7), 19 of which could be quantified (galactaric and galacturonic acids were below the quantitation limit). Compared with the α-dicarbonyls, the contents of carbohydrates in *B. napus* tissues were at least o1 order of magnitude higher and exceeded 100 ng/g f.w. for the five major representatives, glucose, sorbose, sucrose, fructose, and inositol (Fig. 7*A*). The group of low abundance carbohydrates (1–10 nmol/g f.w.) comprised trehalose, rhamnose, and galactinol as well as galactaric, galacturonic, and glucaric acids. Remarkably, although the relative sugar abundances were similar in *A. thaliana*, their absolute contents were at least 7-fold lower in comparison with *B. napus* (Fig. 7*B*).

##### In Vitro Reactivity and Glycative Potential of Plant Sugars

**FIGURE 8.**
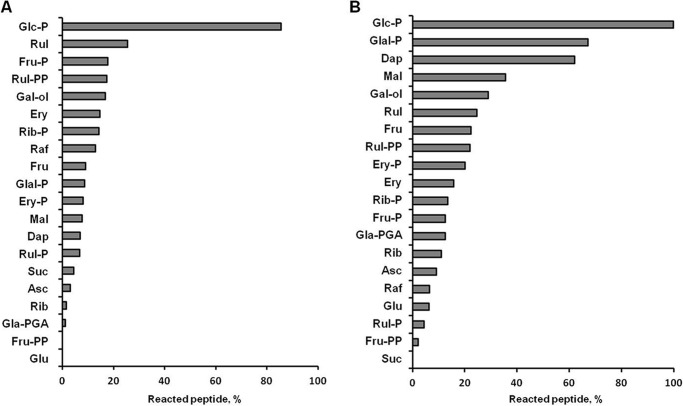
**Relative *in vitro* reactivities of individual carbohydrates (0. 5 mmol/liter) determined in the incubations with equimolar amounts of synthetic peptide Ac-AFGSAKASGA-NH_2_ (*A*) and Ac-AFGSARASGA-NH_2_ (*B*) in 100 mmol/liter sodium phosphate buffer for 42 days at 22 °C while continuously shaking (60 rpm).** The reactivities were determined as the percentage of the consumed peptide by UPLC-ESI-QqTOF-MS operating in the positive ion mode. The peptides were quantified by integration of the characteristic XIC at *m*/*z* 907.46 ± 0.02 and 935.47 ± 0.02, respectively. *Rul-P*, d-ribulose 5-phosphate; *Rul-PP*, d-ribulose 1,5-bisphosphate; *Raf*, d-raffinose; *Rib-P*, d-ribose 5-phosphate; *Ery-P*, d-erythrose 4-phosphate; *Gla-PGA*, d-3-phosphoglyceric acid; *Fru-PP*, d-fructose 1,6-bisphosphate.

**FIGURE 9.**
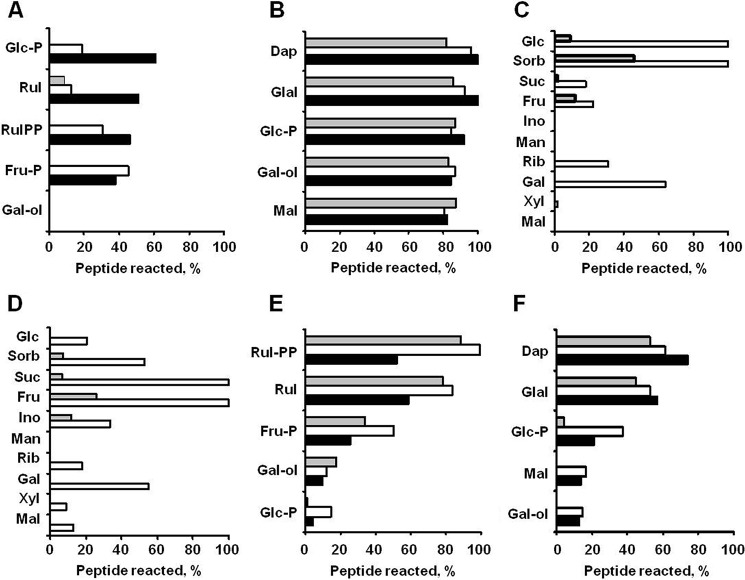
**Relative *in vitro* reactivities of individual carbohydrates toward model synthetic lysyl- (*A*, *C*, and *E*) and arginyl-containing (*B*, *D*, and *F*) peptides determined at 22 °C.** *A* and *B*, 0.5 mm peptides **1** and **2**, respectively, were incubated with the five most reactive carbohydrates at sugar/peptide ratios of 1:10 (*gray*), 1:1 (*white*), and 10:1 (*black*). *C* and *D*, the 10 most abundant plant sugars were incubated with 0.1 mm peptides **1** and **2**, respectively, at relative sugar/peptide ratios corresponding to natural sugar contents derived from Fig. 7 (*white*) or 1:1 (*gray*). *E* and *F*, reactivity of selected sugars (0.5 mm) toward model synthetic peptides (0.5 mm) containing histidine (*gray*), serine (*white*), and glutamic acid (*black*) in the *i* + 4 position relative to the glycation site. *Rul-PP*, d-ribulose 1,5-bisphosphate; *Sorb*, sorbose; *Ino*, inositol; *Xyl*, d-xylose.

To address the ability of the identified potential plant-related glycation agents (*i.e.* carbohydrates) to modify proteins under the conditions mimicking plant growth, we assessed their relative reactivity and glycative potential in well established peptide-based model glycation systems (7, 34). The reactivities of the individual carbohydrates were estimated as consumption of peptides in corresponding peptide-sugar incubations, quantified by integration of the XICs at *m*/*z* 454.23 ± 0.02 and 468.24 ± 0.02, respectively (Table 2). Monosaccharides showed the highest reactivity with peptides **1** and **2** (Fig. 8). However, the reactivity toward **2** was much higher than toward **1**: for peptide **2**, consumption of more than 10% was observed with 15 compared with nine sugars, respectively. Specifically, Glal-P, Dap, and Mal were highly reactive toward the arginyl residue (40–70% of peptide consumption), whereas Glc-P, Rul, d-ribulose 1,5-bisphosphate, Fru-P, and Gal-ol demonstrated similar reactivities with both model sequences (15–25% consumption). For lysine-containing peptide **1**, these values were typically lower and higher when sugar/peptide ratios of 1:10 and 10:1, respectively, were used, whereas only a negligible effect of the sugar/peptide ratio was observed for peptide **2** (Fig. 9, *A* and *B*). In most cases, no linearity was observable between the amount of sugar added and the decrease in initial compound abundance. However, kinetics of sugars, peptides, and the products of their reactions were beyond the scope of this study. The mechanistic and kinetic aspects of these model reactions are being currently studied in detail^4^ and will be published elsewhere.

**TABLE 2:** **Early and advanced glycation products annotated in in vitro glycation mixtures** Incubations were performed for 42 days at 22 °C under continuous shaking; analysis was performed by UPLC-QqTOF-MS operating in the positive ion mode. CMA, *N*^ϵ^-carboxymethylarginine; ArgPyr, argpyrimidine; GLAP, glyceraldehyde-derived pyridinium compound. MGD-HI, methylglyoxal-derived dihydroxyimidazolidine; GD-HI, glyoxal-derived dihydroxyimidazolidine.

Label	Product*^a^*	Reaction partner*^b^*	*m/z*[M + 2H]^2+^_obs_*^c^*	*t_R_*	Residue in the position 6	Δ*m*
				*min*		
**1-1**	Ac-AFGSAKASGA-NH_2_		454.2316	1.9	Lysine	
**1-2**	Ac-AFGSA[pyrraline]ASGA-NH_2_	Ery	574.2633	1.9	5-(Hydroxymethyl)-1*H*-pyrrole-2-carbaldehyde	108.021
**1-3**	Ac-AFGSA[GLAP]ASGA-NH_2_	Glal-P	589.7723	2.0	1- (5-Acetylamino-5-carboxypentyl)-3-hydroxy-5-hydroxymethylpyridinium	109.0284
**1-4**	Ac-AFGSA[CEL]ASGA-NH_2_	Dap	490.2422	2.8	*N*^ϵ^-Carboxyethyllysine	72.0211
**1-5**	Ac-AFGSAK_[triose]_ASGA-NH_2_	Dap	490.2422	2.0	*N*-Triosyllysine	72.0212
**1-6**	Ac-AFGSA[CML]ASGA-NH_2_	Rul	483.2344	2.6	*N*^ϵ^-Carboxymethyllysine	58.0055
**1-7**	Ac-AFGSAK_[hexose]_ASGA-NH_2_	Fru	535.2581	1.8	*N-*Hexosyllysine	162.0529
**1-8**	Ac-AFGSAK_[pentose]_ASGA-NH_2_	Rib	520.2528	1.9	*N-*Pentosyllysine	132.0423
**1-9**	Ac-AFGSAK_[tetrose]_ASGA-NH_2_	Fru	505.2475	1.9	*N-*Tetrosyllysine	102.0317
**2-1**	Ac-AFGSARASGA-NH_2_		468.2347	2.0	Arginine	
**2-2**	Ac-AFGSA[Glarg]ASGA-NH_2_	Ery	488.2322	2.0	1-(-4-Amino-4-carboxybutyl)2-imino-5-oxoimidazolidine	39.9949
**2-3**	Ac-AFGSA[MG-H]ASGA-NH_2_	Dap	495.2400	2.3	Methylglyoxal-derived hydroimidazolone	54.0105
**2-4**	Ac-AFGSA[CMA/GD-HI]ASGA-NH_2_	Rul	497.2375	2.4	*N*^δ^-Carboxymethylarginine/*N*^δ^- (3.4-dihydroxy-1-imidazolidin-2-yl)ornithine	58.0055
**2-5**	Ac-AFGSA[CEA/MGD-HI]ASGA-NH_2_	Dap	504.2453	2.6	*N*^δ^-Carboxyethylarginine/2-amino-5-(4,5-dihydroxy-5-methyl-4,5-dihydro-1*H*-imidazol-2-ylamino)pentanoic acid	72.0211
**2-6**	Ac-AFGSA[ArgPyr]ASGA-NH_2_	Glal-P	508.2478	3.2	*N*^δ^-(5-Hydroxy-4,6-dimethylpyrimidine-2-yl)-l-ornithine	80.0262

*^a^* The analytes are listed in the order of their retention times. AGE-modified lysyl and arginyl residues are marked with brackets; Amadori or Heyns products on lysyl (K) residues are subscript with brackets.

*^b^* Abbreviation of the sugar used in the incubation where annotation of the analyte was performed.

*^c^* All analytes were annotated with mass accuracy ≤3ppm; XICs were calculated for *m*/*z* ± 0.02.

To address the question whether less reactive, but highly abundant sugars can result in higher glycation rates, we incubated peptides **1** and **2** with the 10 most abundant plant carbohydrates taken in their relative natural content ratios (Fig. 7), which were set from 1:1 for Mal to 792:1 for Glc as described under “Experimental Procedures.” Indeed, the majority of the tested carbohydrates were potent glycation agents under high sugar/peptide ratios (Fig. 9, *C* and *D*). Thus, incubation of **1** in presence of a high excess of Glc and sorbose resulted in its complete consumption, whereas the same was observed for **2** in the presence of high Suc and Fru concentrations. Additional experiments revealed the influence of neighboring residues (in the *i* + 4 position relative to the glycation site) on peptide reactivity. However, although the basic residue (histidine) typically increased the reactivity, the effect of an anionic amino acid (glutamic acid) varied for different sugars (Fig. 9, *E* and *F*). Remarkably, as can be seen for Gal-ol and Glc-P, the relative effects of neighboring residues were similar for both lysyl-and arginyl-containing sequences.

The glycation potential of the individual sugars was estimated by the relative abundances of the specific glycation products (Tables 3 and 4). Thus, in these *in vitro* experiments, we estimated the potential ability of selected plant-derived sugars to form specific glycation products, previously annotated in *B. napus* plants. Depending on the structure of the generated products, early and advanced glycation potential could be quantitatively assessed as the relative abundances of corresponding analytes (*i.e.* Amadori/Heyns compounds or individual AGEs). In this context, Dap, Ery, Fru, and Fru-P were the most potent carbohydrate precursors of the triose-, tetrose-, pentose-, and hexose-derived Amadori and/or Heyns compounds (**1-5**, **1-9**, **1-8**, and **1-7**), respectively (Table 3). The lysine-derived AGEs (CML, glyceraldehyde-derived pyridinium compound, and pyrraline) originated preferably from Rul, Suc, and Gla, respectively, whereas Dap, Rul, Glal-P, and Ery were the most potent precursors of the arginine-derived AGEs, *i.e.* compounds **2-2**, **2-3**, **2-4**, **2-5**, and **2-6** (Tables 3–4).

**TABLE 3:** ***In vitro* potential of individual carbohydrates for early and advanced glycation by lysine residues** The incubations were performed with 0.5 mm synthetic peptide Ac-AFGSAKASGA-NH_2_ and 0.5 mm carbohydrate in 100 mmol/liter sodium phosphate buffer for 42 days under 22 °C and continuous shaking (60 rpm). GLAP, glyceraldehyde-derived pyridinium compound. Tri, Tetr, Pent, Hex, triose-, tetrose-, pentose-, and hexose-derived early glycated (Amadori/Heyns products); Pyr, pyrraline; b.d.l., specific product is below detection limit; Fru-PP, d-fructose 1,6-bisphosphate; Raf, d-raffinose; Rib-P, d-ribose 5-phosphate; Rul-P, d-ribulose 5-phosphate; Rul-PP, d-ribulose 1,5-bisphosphate; Ery-P, d-erythrose 4-phosphate; Gla-PGA, d-3-phosphoglyceric acid.

Glycation product	*m/z*	Carbohydrate glycation potential*^a^*																			
		Asc	Dap	Ery	Ery-P	Fru	Fru-P	Fru-PP	Gal-ol	Glc	Glc-P	Glal-P	Gla-PGA	Mal	Raf	Rib	Rib-P	Rul	Rul-P	Rul-PP	Suc
		%																			
Tri/CEL	979.48	2	**100***^b^*	b.d.l.	4	1	7	1	b.d.l.	b.d.l.	b.d.l.	90	b.d.l.	b.d.l.	b.d.l.	1	6	1	11	30	b.d.l.
Tetr	1009.49	16	b.d.l.	**100***^b^*	5	21	48	b.d.l.	b.d.l.	3	b.d.l.	b.d.l.	b.d.l.	b.d.l.	4	11	3	8	b.d.l.	9	b.d.l.
Pent	1039.51	b.d.l.	b.d.l.	b.d.l.	b.d.l.	**100***^b^*	52	b.d.l.	b.d.l.	b.d.l.	b.d.l.	b.d.l.	b.d.l.	b.d.l.	b.d.l.	42	b.d.l.	14	b.d.l.	63	b.d.l.
Hex	1069.52	b.d.l.	b.d.l.	b.d.l.	b.d.l.	17	**100***^b^*	b.d.l.	b.d.l.	70.	81	b.d.l.	b.d.l.	61	61	b.d.l.	b.d.l.	b.d.l.	b.d.l.	b.d.l.	b.d.l.
CML	965.47	24	27	73	47	31	75	1	b.d.l.	2	b.d.l.	71	b.d.l.	b.d.l.	b.d.l.	88	26	**100***^b^*	19	13	1
GLAP	1178.54	40	b.d.l.	23	b.d.l.	b.d.l.	b.d.l.	55	63	20	b.d.l.	**100***^b^*	7	26	9	16	59	26	41	b.d.l.	18
Pyr	1147.52	14	b.d.l.	**100***^b^*	19	32	70	46	b.d.l.	b.d.l.	b.d.l.	9	b.d.l.	b.d.l.	b.d.l.	59	22	10	73	b.d.l.	b.d.l.

*^a^* Carbohydrate glycation potential was estimated by characteristic signal intensities.

*^b^* For each glycation product, signal intensities (*i.e.* peak areas calculated by integration of corresponding XICs, *m*/*z* ± 0.02, at characteristic *t_R_*) observed with individual carbohydrates were referred to those obtained with the most reactive one (100%; bold).

**TABLE 4:** **In vitro potential of individual carbohydrates for advanced glycation by arginine residues** The incubations were performed with 0.5 mmol/liter synthetic peptide Ac-AFGSARASGA-NH_2_ and 0.5 mm carbohydrate in 100 mm sodium phosphate buffer for 42 days under 22 °C and continuous shaking (60 rpm). CMA, *N*^ϵ^-carboxymethylarginine; ArgPyr, argpyrimidine; b.d.l., specific product is below detection limit; Fru-PP, d-fructose 1,6-bisphosphate; Raf, d-raffinose; Rib-P, d-ribose 5-phosphate; Rul-P, d-ribulose 5-phosphate; Rul-PP, d-ribulose 1,5-bisphosphate; Ery-P, d-erythrose 4-phosphate; Gla-PGA, d-3-phosphoglyceric acid.

AGE	*m/z*	Carbohydrate glycation potential*^a^*																			
		Asc	Dap	Ery	Ery-P	Fru	Fru-P	Fru-PP	Gal-ol	Glc	Glc-P	Glal-P	Gla-PGA	Mal	Raf	Rib	Rib-P	Rul	Rul-P	Rul-PP	Suc
		%																			
CEA	1007.49	3	**100***^b^*	1	16	b.d.l.	b.d.l.	5	b.d.l.	b.d.l.	b.d.l.	87	b.d.l.	b.d.l.	b.d.l.	b.d.l.	7	1	7	11	b.d.l.
CMA	993.47	73	3	81	20	9	7	b.d.l.	b.d.l.	1	b.d.l.	15	b.d.l.	b.d.l.	b.d.l.	19	6	**100***^b^*	6	1	b.d.l.
ArgPyr	1015.49	b.d.l.	72	b.d.l.	b.d.l.	b.d.l.	b.d.l.	b.d.l.	b.d.l.	b.d.l.	b.d.l.	**100***^b^*	b.d.l.	b.d.l.	b.d.l.	b.d.l.	b.d.l.	b.d.l.	b.d.l.	b.d.l.	b.d.l.
Glarg	975.46	79	b.d.l.	**100***^b^*	27	13	19	b.d.l.	b.d.l.	b.d.l.	b.d.l.	26	b.d.l.	b.d.l.	32	25	24	77	24	b.d.l.	5
MG-H	989.48	3	**100***^b^*	b.d.l.	9	b.d.l.	4	b.d.l.	b.d.l.	b.d.l.	b.d.l.	84	b.d.l.	b.d.l.	b.d.l.	b.d.l.	7	2	7	25	b.d.l.

*^a^* Carbohydrate glycation potential was estimated by characteristic signal intensities.

*^b^* For each glycation product, signal intensities (*i.e.* peak areas calculated by integration of corresponding XICs, *m*/*z* ± 0.02, at characteristic *t_R_*) observed with individual carbohydrates were referred to those obtained with the most reactive one (100%; bold).

## Discussion

### 

#### 

##### Plant Experiments

In the characterization of plant protein glycation, we addressed both reproducibility and specificity of AGE patterns in a plant proteome. For these experiments, *B. napus* was selected as a convenient tool for optimizing our two-dimensional chromatography (LC × LC) proteomic approach (previously well established in human proteomics (40)) for plants as this species produces much biomass, and hence sufficient amounts of protein required for BAC could be easily obtained. Moreover, this species is a close relative of *A. thaliana* whose genome is completely sequenced. Hence, a reliable database search and cross-validation with *A. thaliana* could be done in the next step to address the species specificity of the observed glycation patterns.

Thus, in the first step, we characterized the constitutive glycation patterns, *i.e.* those normally appearing during the plant development. To provide the equal mineral nutrition and air supply conditions for all plants and to exclude growth differences related to the heterogeneity of substrate structure, we grew the plants in an aqueous system well established in our laboratory. To address the reproducibility of AGE patterns, we performed our experiment twice under identical growth conditions and considered the proteins found modified in both replicates as constitutively glycated (with respect to constant environmental conditions). We found two replicates sufficient due to the high reproducibility of established and well controlled growth conditions. Performing two experiments would allow addressing the effects of other factors (*e.g.* seed sterilization and fluctuations of water quality).

As was clearly demonstrated by Bechtold *et al.* (33), the levels of protein glycation were increased upon exposure of plants to environmental changes. As these changes might result in the development of oxidative stress, characterization of the plant oxidative status in both experimental replicates was of major importance. As polyunsaturated fatty acid residues in lipids are sensitive targets for ROS attack, the lipid hydroperoxide content can be considered as the most accurate indicator of oxidative stress (43). In this context, the absence of an inter-replicate difference in this parameter (Fig. 2*A*) clearly indicated the same oxidative status of the both plant groups, and this was additionally confirmed by similar Asc/DHA and GSH/GSSG ratios (Fig. 2, *B* and *C*). Thus, all plants had the same tissue levels of ROS and antioxidants and could be expected to produce similar constitutive glycation patterns.

##### Plant AGE-modified Proteome

The study of the AGE-modified proteome is a challenging task. Due to the low abundances of individual glycated peptides, the corresponding signals are rarely selected for MS/MS by the DDA algorithm in conventional shotgun LC-MS experiments (13). This so-called analytical “undersampling” is a well known disadvantage of the DDA algorithm (12). It typically results in lower numbers of annotated proteins, reduced sequence coverage, and a complete lack of the low abundance polypeptides. Unfortunately, the high structural heterogeneity of AGEs makes enrichment/depletion protocols inapplicable for their analysis. However, the introduction of a gas phase fractionation approach and a *t_R_*-dependent exclusion of unmodified peptides from fragmentation (*i.e.* reanalysis of the samples with a DDA-based exclusion of selected peptide signals from the MS/MS fragmentation using a so-called “exclusion list” integrated in the instrumental method) essentially increases the rates of identified glycated peptides (38). We assume that this strategy might be a good alternative to prefractionation and LC × LC-based techniques (44) that are often time-consuming and complex in data interpretation.

In both *B. napus* and *A. thaliana*, AGE modification sites were dominated by arginine-derived modifications (Fig. 4, *A* and C), corresponding well to the data obtained with humans by LC-MS/MS-based amino acid analysis (45). This distribution can be generally explained by the amino acid composition of the proteins found to be glycated. Indeed, the arginine contents in those polypeptides reached 23% of the sequence, in 60% of the cases being more abundant than lysine (up to a 10-fold difference). Unexpectedly, although the tissue content of MGO was 2.5–3-fold lower in comparison with GO, the number of MGO-derived modification sites was higher in both plants (Fig. 4, *B* and *D*). Most probably, the half-life times of the MGO-derived hydroimidazolones were longer under physiological conditions. This could be explained by both their relatively higher stability and lower reactivity, for example, toward amino acids and polyamines. Obviously, the interaction with lysyl and arginyl residues depends on the molecular surrounding (7), *i.e.* the sequence and tertiary structure of each specific protein. This influence can be direct (*i.e.* via interaction with neighboring residues) or mediated by the stability of the corresponding adducts.

The fact that advanced glycation levels of *B. napus* proteins were 50% higher in comparison with *A. thaliana* (1082 *versus* 692 AGE-modified sites) can be explained by its stronger antiglycative enzymatic defense. Indeed, the levels of MGO and GO were lower in *A. thaliana*, whereas the glyoxalase II activity was approximately 2.5 times higher. Taking into account that most of the glycation sites were MGO-derived, this factor might be essential in the observed species-specific difference in advanced glycation patterns. Most probably, the lower level of antiglycative defense in *A. thaliana* is underlied by the lower sugar contents in its tissues.

The distribution of AGE-modified proteins by functional groups was rather similar in both plants. Generally, the polypeptides involved in metabolism of proteins and nucleic acids were the preferable targets of advanced glycation: their relative glycation rates exceeded their relative impact in the proteome, and this was more pronounced for *B. napus* (Table 1). This can be explained by the overall expression levels of corresponding genes, amino acid composition, structure, and functional aspects.

Thus, essential involvement of the polypeptides interacting with DNA in advanced glycation can be explained by the relatively high number of well accessible arginyl residues on their surface (46). These residues are typically involved in binding to negatively charged DNA. Indeed, the sequence analysis of our data sets revealed ∼1.5-fold higher contents of arginyl residues (in comparison with lysyl residues) in such proteins. The modifications formed as well as AGE-related protein-DNA cross-links might be an important factor impacting the regulation of transcription. Intensive AGE modification of polypeptides related to protein metabolism might indicate the role of glycation in aging (47). As has been learned from experiments with mammalian cell cultures, glycation affects the rates of protein degradation (48). Accordingly, 11 and 60 polypeptides involved in protein synthesis and degradation, respectively, were found to be AGE-modified in this study. Essential involvement of stress-related proteins and elements of antioxidative and antiglycative defense in advanced glycation raises questions about (i) possible regulatory role of such modifications (*i.e.* their influence on activities of defense enzymes and/or expression of related genes) and (ii) involvement of glycation in aging and the environmental stress response. Therefore, in future work, the relation of this specific glycation to aging and environmental stress needs to be addressed.

The most striking difference observed here in comparison with all reports dealing with the patterns of advanced glycated proteins in mammals is the number of identified AGE-modified sites. Indeed, here we report 772 AGE-modified proteins, whereas only several dozen of *in vivo* advanced glycation sites have been described in mammals so far. Recently, Greifenhagen *et al.* (12) identified 11 carboxymethylated lysyl residues in five human plasma proteins using a combination of CML-specific precursor ion scanning and targeted DDA experiments. Applying the same approach to the MG-H and glyoxal-derived hydroimidazolone residues, Schmidt *et al.* identified a further 45 AGE-modified human plasma polypeptides containing up to three glycation sites each (13). Using a proteomic approach, Rabbani and Thornalley (46) reported several hot spot modification sites in endothelial proteins. The higher involvement of the plant proteome in AGE formation can be explained by a different (in comparison with animal or human systems) metabolic background characterized by a higher reactivity toward proteins and a stronger glycation potential. Indeed, the metabolically active tissues of plants are characterized by rich patterns of sugar-related metabolites (Fig. 7). Thus, relatively high levels of pentoses, tetroses, and trioses as well as the presence of their phosphorylated analogs can be detected in plant systems. This might dramatically affect the mechanisms and pathways of protein glycation in plants and make them quite different from those in mammals.

##### Precursors and Pathways of AGE Formation in Plants

In this context, knowledge about the metabolome background is the prerequisite for the understanding of the *in vivo* mechanisms of protein glycation. Generally, there are two principle pathways of advanced glycation (Fig. 1): glycoxidation, *i.e.* formation of AGEs via oxidative degradation of Amadori and Heyns products (49) or Schiff bases (Namiki pathway) (50), and autoxidative glycosylation, *i.e.* AGE formation via α-dicarbonyl intermediates without formation of early glycation products (49). Both pathways of AGE formation have been observed *in vivo* in mammalian tissues (51) and during thermal processing of foods (52). To address the role of the principle pathways of advanced glycation in plants, we considered the possible precursors of AGEs.

Surprisingly, the numbers of early and advanced glycated sites (as well as their ratio) observed here differed drastically from the results obtained for the human blood proteome (currently the only well studied glycated proteome). Thus, thousands of early glycated proteins could be identified by the combination of BAC and nano-LC-MS/MS DDA in plasma and erythrocyte membranes of diabetic patients (11). Although the same strategy was applied here, the number of the identified glycation sites was limited to less than 200. Remarkably, although the distribution of the identified Amadori/Heyns compounds favored products of interaction with highly reactive trioses, tetroses, and pentoses (Fig. 6*A*), none of the early glycated sequences were found to be AGE-modified, indicating a negligible impact of glycoxidation in formation of AGEs in plants. Thus, most probably, carboxymethylation and carboxyethylation sites identified here originated from GO and MGO, respectively, rather than from Amadori and Heyns compounds. The low number of identified early glycation sites clearly indicates the presence of potent deglycation mechanisms in plants. The enzyme ribulosamine/erythrulosamine 3-kinase characterized by Fortpied *et al.* (26) in spinach (*Spinacia oleracea*) and *A. thaliana* is the most probable candidate for such a deglycation system: in spinach, it was 700 times more active than the fructosamine 3-kinase from human erythrocytes. Thus, this factor could suppress the glycoxidative pathway in plants.

In this context, autoxidative glycosylation seems to be the major source of the plant AGEs. To address the question concerning the sugars involved, we performed incubations with model synthetic peptides under conditions simulating the plant growth. Our observations generally supported the published data. The high reactivity of triose phosphates and Glu-P (Fig. 8) was reported previously for *in vitro* protein glycation studies, although the authors could not identify the products formed from each individual metabolite (53). However, to the best of our knowledge, the high reactivity of Rul, Ery, and the related sugar phosphates was unambiguously confirmed here for the first time (Fig. 8). Interestingly, although the reactivities of Rib, Fru, Suc, and Glc were lower due to their high tissue contents (Fig. 7), they turned out to be potent glycation agents (Fig. 9, *C* and *D*). In this context, they might also be the major precursors of the early glycation products and the reason for the high levels of ribulosamine/erythrulosamine 3-kinase activity reported previously (26). Remarkably, the fact that reserve and transport sugars (Mal, Raf, and Gal-ol) demonstrated essential reactivities toward **1** and **2** (Fig. 8) makes plant protein modifications even more probable. In our recent work, we addressed the effects of neighboring amino acid residues on the formation and stability of AGEs and clearly demonstrated that, in the case of Glc-dependent glycation, basic residues catalyze AGE formation, whereas the acidic residues have an opposite effect (7). However, the results obtained here with the same peptide sequences (**3**-**8**) (selected to ensure reliable comparison with previous results) and a broad panel of sugars indicate that this reactivity is dependent on the nature of the glycation agent as well.

As far as the AGEs are concerned, formation of CML from Rib and Fru (54) was confirmed in our study (Table 3), whereas Rul turned out to be an even more important factor in its generation. The formation of MGO-derived modifications (Table 4) from Dap (MG-H and CEA) or Glal-P (argpyrimidine) supports the previous reports regarding the role of triose phosphates as the *in vivo* source of MGO (46). Accumulation of GO-derived modifications in the incubations with Ery and Rul was in accordance with their high peptide reactivities (Fig. 8). Although, according to our results, the formation of such AGEs in plants occurs most likely via the autoxidative pathway, the role of this mechanism in CML formation *in vitro* is still to be confirmed. Generally, it is clearly seen that the most potent precursors of plant AGE are the metabolites of glycolysis and the Calvin cycle (Tables 3 and 4). Due to phosphorylation, these intermediates are highly reactive and present in cells in small equilibrium concentrations, and the rates of their production change essentially depending on the time of day. The fact that most of these sugars are more reactive than glucose (Fig. 8) indicates that they can be easily involved in metal-catalyzed monosaccharide autoxidation (55) and that the levels of their generation are much higher in plants than in mammals. This result is in agreement with the AGE patterns found here (Fig. 4).

##### Conclusions

To the best of our knowledge, a comprehensive study of the plant AGE proteome is presented here for the first time. Surprisingly and in contrast to the well characterized human plasma proteome, the levels of early glycation in *B. napus* were limited, whereas the number of AGE modification sites was 2–3 orders of magnitude higher than detected in human plasma and tissues. Most probably, this striking difference was caused by the high activity of enzymatic antiglycative defense, specific qualitative and quantitative patterns of potential glycation agents, and hence other glycation mechanisms. Thus, the impact of early glycation and hence glycoxidation in overall AGE formation is negligible. Rather, the majority of AGEs is generated via the autoxidative pathway. *In vitro* assay of the plant-specific glycation reactions clearly suggested fructose, ribose, ribulose, erythrose, and sugar phosphates (constantly generated in metabolically active tissues) as the most likely precursors of the plant AGEs observed.

## Author Contributions

E. L., D. B., U. G., J. M., A. F., C. M., and N. O. designed and performed plant experiments and stress characterization. E. L., T. B., and A. F. designed and performed proteomic experiments. T. B., E. T., G. P., and C. B. designed and performed metabolomic experiments. U. G., E. L., N. F., G. U. B., and A. F. performed *in vitro* experiments with model peptides. E. L., C. M., A. T., C. B., T. V., L. A. W., and A. F. interpreted data and wrote the manuscript.

## Footnotes

4 A. Frolov *et al.*, unpublished data.

## Footnotes

3 The abbreviations used are:

AGE: advanced glycation end product
aq.: aqueous
Asc: l-ascorbate
BAC: boronic acid affinity chromatography
CEA: *N*^δ^-(carboxyethyl)arginine
CEL: *N*^ϵ^-carboxyethyllysine
CML: *N*^ϵ^-carboxymethyllysine
Dap: dihydroxyacetone phosphate
DDA: data-dependent acquisition
DHA: dehydroascorbic acid
Ery: d-erythrose
ESI: electrospray ionization
Fmoc: 9-fluorenylmethoxycarbonyl
Fru-P: d-fructose 6-phosphate
f.w.: fresh weight
Gal-ol: galactinol dehydrate
Glal-P: d-glyceraldehyde 3-phosphate
Glc-P: d-glucose 6-phosphate
GO: glyoxal
LIT: linear ion trap
Mal: d-maltose
MG-H: methylglyoxal-derived hydroimidazolone, *N*^δ^-(5-methyl-4-oxo-5-hydroimidazolinone-2-yl)-l-ornithine
MGO: methylglyoxal
Orbitrap: orbital trap mass analyzer
ROS: reactive oxygen species
Rubisco: ribulose bisphosphate carboxylase/oxidase
Rul: d-ribulose
Suc: d-sucrose
*t_R_*: retention time
QqTOF: quadrupole-time of flight
UPLC: ultrahigh-performance liquid chromatography
XIC: extracted ion chromatogram.
